# The effect of the 2018 Japan Floods on cognitive decline among long-term care insurance users in Japan: a retrospective cohort study

**DOI:** 10.1186/s12199-021-01038-9

**Published:** 2021-12-02

**Authors:** Shuhei Yoshida, Saori Kashima, Masatoshi Matsumoto

**Affiliations:** 1 Department of Community-Based Medical System, Graduate School of Biomedical and Health Sciences, Hiroshima University, 1-2-3 Kasumi, Minami-ku, Hiroshima-shi, Hiroshima-ken, 734-8551 Japan; 2 Environmental Health Sciences Laboratory, Graduate School of Advanced Science and Engineering, Hiroshima University, 1-3-2 Kagamiyama, Higashi-Hiroshima-shi, Hiroshima-ken, Japan

**Keywords:** Natural disaster, Disaster preparedness, Long-term care, Claim data, Cognitive decline

## Abstract

**Background:**

The July 2018 Japan Floods caused enormous damage to western Japan. Such disasters can especially impact elderly persons. Research has shown that natural disasters exacerbated a decline in cognitive function, but to date, there have been no studies examining the effects of this disaster on the elderly. The object of this study was to reveal the effect of this disaster in terms of cognitive decline among the elderly.

**Methods:**

Study participants were certified users of the long-term care insurance (LTCI) system in Hiroshima, Okayama, and Ehime prefectures from May 2018 to June 2018. The observation period was from July 2018 to December 2018. Our primary outcome was cognitive decline after the disaster using a dementia symptomatology assessment. In addition to a crude model, a multivariate Cox proportional hazards model was used to assess the cognitive decline of victims, adjusting for age classification, gender, the level of dementia scale before the disaster occurred, residential environment, whether a participant used facilities shut down after the disaster, and population density. After we confirmed that the interaction term between victims and residential environment was statistically significant, we stratified them for the analysis.

**Results:**

The total number of participants was 264,614. Victims accounted for 1.10% of the total participants (*n* = 2,908). For the Cox proportional hazards model, the hazard ratio of the victims was 1.18 (95% confidential interval (CI): 1.05–1.32) in the crude model and 1.12 (95% CI: 1.00–1.26) in the adjusted model. After being stratified by residential environment, the hazard ratio of home victims was 1.20 (95% CI: 1.06–1.36) and the hazard ratio of facility victims was 0.89 (95% CI: 0.67–1.17).

**Conclusions:**

This study showed that elderly living at home during the 2018 Japan Floods were at risk for cognitive decline. Medical providers, care providers, and local governments should establish a system to check on the cognitive function of elderly victims and provide necessary care support.

## Background

Torrential rains in western Japan between 28 June and 8 July 2018 caused enormous damage that affected local residents [1, 2]. The rains were named the July 2018 Japan Floods (*2018-nen-sitigatu-gou*) by the Japan Meteorological Agency [3]. The impact included 237 fatalities, eight missing, 433 injured, and 6767 houses completely destroyed [4]. Because transportation networks and utilities were also damaged, necessary emergency relief supplies were often unable to be delivered to damaged areas. The magnitude of disaster damage was the second largest in the twenty-first century, second to the Great East Japan Earthquake (GEJE) [5].

Past studies have shown that victims of natural disasters suffer from a variety of health problems [6]. In addition to the direct deaths caused by disasters, there are various impacts on physical or mental health outcomes after disasters [7–9]. Moreover, elderly people are vulnerable to disasters. The majority of the victims and impacts occur among elderly people [5]. This was also the case in the 2018 Japan Floods, and 90% of the victims were elderly people over 65 years old. Therefore, it is important to develop specific countermeasures for this group, especially as heavy torrential rains occur almost every year in Japan [10].

In addition to high mortality rates, previous studies have shown an increased risk of cognitive decline and dementia among the elderly after natural disasters. Studies of GEJE, Hurricane Katrina in the USA, and Hurricane Maria in Puerto Rico have suggested that these natural disasters influence dementia and cognitive decline [11–16]. In particular, cognitive decline was observed not only in the immediate aftermath of the GEJE, but also over the long term [17, 18]. A similar trend in long-term effects was also reported after the Chuetsu Earthquake in Japan [19]. Furthermore, regional characteristics, such as rurality, can generally affect cognitive decline [20]. However, since the participants of previous studies were limited to only damaged municipalities or facilities, there have been no studies to evaluate the decline in cognitive function of the whole population after a natural disaster.

In Japan, a long-term care insurance (LTCI) system was introduced in 2000 [21–23]. The LTCI system provides various care services to mainly the elderly who have a decline in activities of daily living (ADL) or cognitive function. The care level is certified in accordance with the amount of care needed as determined by care professionals and primary care physicians. When judging care level, cognitive function must be evaluated. Based on the result of this certification, the elderly can use LTCI services.

Japan has the highest aging rate in the world. Over five million people are included with certification of their care level, because almost all elderly who need care use the LTCI service. Therefore, the LTCI system evaluates changes in cognitive function of almost all elderly who need care and who were impacted by the 2018 Japan Floods. The aim of this study was to reveal the effect of this disaster on the cognitive decline of vulnerable elderly. We also discuss future disaster preparedness in an aging society.

## Methods

### Study design

This study was a retrospective cohort study.

### LTCI system

**Figure 1:**
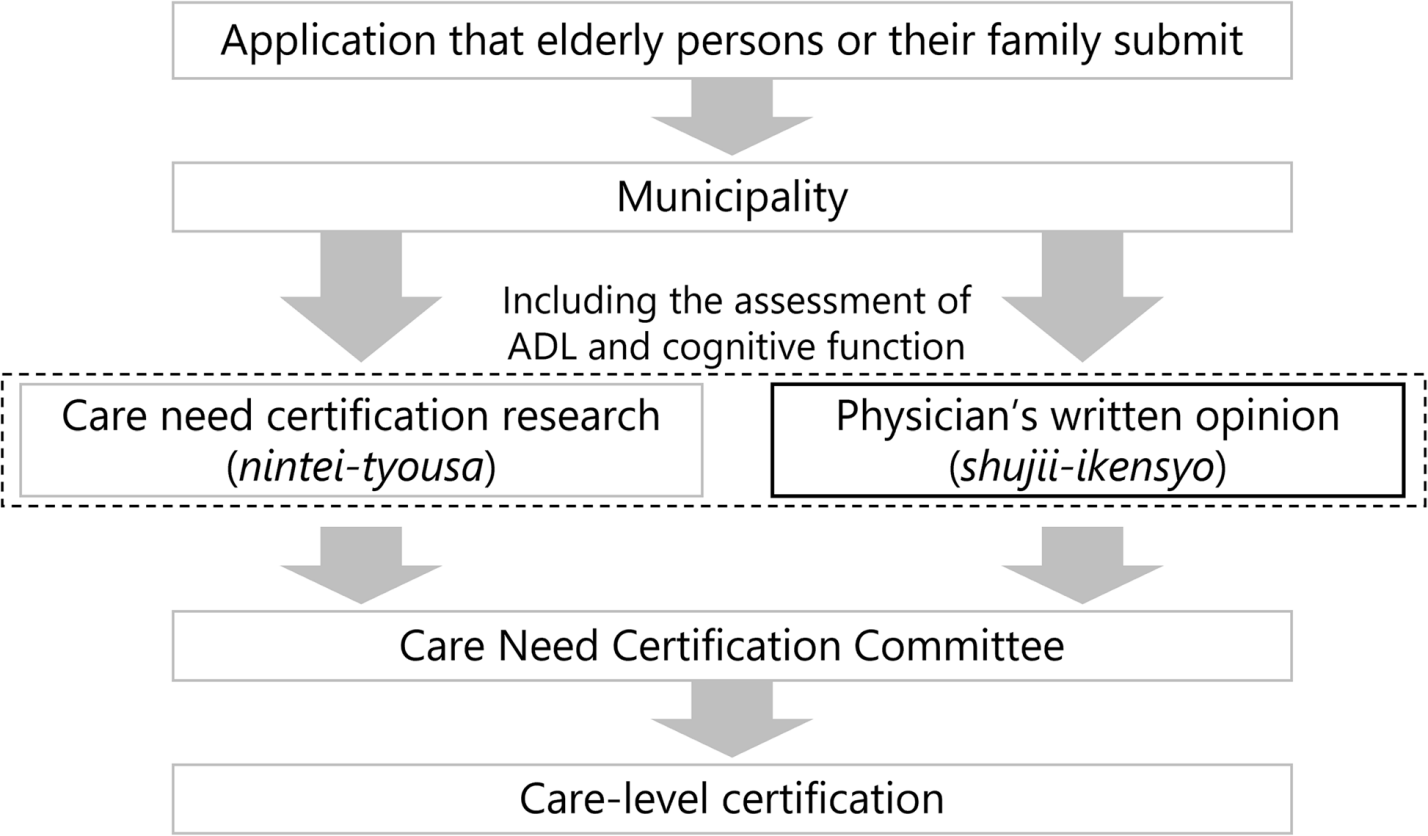
The flow of care-level certification. ADL, activity of daily living

The use of LTCI services requires certification of care level. After an application is submitted by the elderly persons themselves or a family member, the municipality, as the insurer, orders two investigations for the applicant (Fig. 1). One of the investigations is a care need certification (*nintei-tyousa*), which is a visit by a care-related professional and an evaluation of their care needs by using a structured questionnaire. The other is a physician’s written opinion (*shujii-ikensyo*), which is a care evaluation by a primary care physician. Through the evaluation, a primary care physician scores activities of daily living and cognitive function by using a structured scale common to the whole country. Based on both investigations, a Care Need Certification Committee determines the care level of applicants. The care levels are divided into seven levels (support need levels 1–2 and care need levels 1–5). The higher the care level, the more services that are available to a certified person in a month. If the care needs vary due to a change of ADL or cognitive function, applicants can apply for a re-certification of care level. Out-of-pocket expenditures for LTCI users range from 10 to 30% according to income.

LTCI services are roughly divided four types: (1) home-visit services providing care by nurses, rehabilitators, or assistants in their private homes, (2) day services providing care in facilities during the day by rehabilitators or assistants, (3) short-stay services that consist of respite care for a short period, and (4) facility services that provide residence care to those who are unable to live at home. Re-certification is done when a disease progresses or there is a decline of ADL or cognitive function that requires increased services. Generally, users requiring a certain high care level are admitted to a facility.

### Data on LTCI users

This study was conducted using a special sampling of certification data for long-term care and LTCI claim data (approval no. 0711-1). These data were stored in a LTCI comprehensive database which is administered by the Ministry of Health, Labour and Welfare (MHLW). This database maintains digitized claims of LTCI summarized monthly with details for all services used by each user. The MHLW has provided datasets extracted from this database for research institutes since 2018 based on expert council approval.

### Data on municipalities

We compared the rurality of the residential area of LTCI users. Using the data on population and land area in each municipality published by the Statistics Bureau, Ministry of Internal Affairs and Communications, the population density of the municipality was collated with individual-level information [24].

### Setting

The setting was Hiroshima, Okayama and Ehime prefectures. The worst damage from this disaster occurred in these prefectures: 212 out of 237 deaths, 8 out of 8 missing, 6603 out of 6767 houses completely destroyed, and 10,012 out of 11,243 houses were partially destroyed [5].

### Participants and definition of disaster victims

Participants were certified users of the LTCI system in Hiroshima, Okayama and Ehime prefectures from May 2018 to June 2018. The observation period was from July 2018 to December 2018. Victims were defined as participants who changed to an exempt monthly fee for LTCI services after the disaster, reflecting the announcement by the MHLW that all victims of this disaster were exempt from LTCI service fees. This was applied even if they used LTCI services in another region from their registered home region. Local governments authorized a designation as a victim when a LTCI user’s house was completely or partially destroyed, burned down, or there was flooding of a floor, or similar damage, and/or when a main breadwinner died, became seriously injured, ill, or missing. Few people were exempted from the LTCI service fee prior to the disaster. It was reported that the number of deaths in three prefectures was 81 among people from the age of 20 to 69, who represent those most likely to be employed and the primary breadwinners. The number of missing was eight among all ages [25]. The number of deaths was 38% (81/212) for all deaths among all ages in the three prefectures [5]. In addition, the number of completely unemployed increased by three persons compared to before the disaster [26]. In contrast, there were 16,615 cases of destroyed homes in the three prefectures [5]. Therefore, we estimated the majority of the reasons for certification as a victim were due to home damage. Because this exemption excluded those who paid no out-of-pocket expenditures for the LTCI service fee, such as welfare recipients and A-bomb survivors, they were included in the non-victim group. Among LTCI users in the setting prefectures, there are 3475 people who changed status to become exempted from self-payment after the disaster [27]. Because we identified 3024 victims in this data set, the capture ratio for registration would be 87.0%.

We excluded certified users whose cognitive function were the worst on the rating scale from a physician’s written opinion, because a further cognitive decline could not be assessed.

### Variables

The outcome variable was cognitive decline. A physician’s written opinion (*shujii-ikensyo*) include the dementia symptomatology assessment (DSA) to certificate the care level of LTCI insurance. This is a nationally standardized dementia scale to assess level of independence in cognitive functions (*nintisyou-koureisya-no-seikatu-jiritudo*) [12]. A care need certification examination also uses the same scale to assess dementia symptomatology. Although both investigations were conducted independently, the results showed a high correlation [12]. In addition to this, the DSA was proven to have high inter-rater reliability [28]. The level of dementia scale has high correlation with the Mini Mental State Examination and level I was equivalent to a 0.5 point on the Clinical Dementia Rating [29, 30]. We judged the decline of cognitive function when a primary care physician evaluated the DSA at the point of re-certification and there was a worsened level compared with the before result of DSA during the observation period. The DSA was evaluated with a re-certification of care level in the following cases.

1. The valid certification period of care level ends. The period is generally one year.
2. User applies for recertification of their care level due to worsening of their disease or increase in total amount of care needed.

We adopted the following variables as potential confounders: age classification, gender, the level of dementia scale before the disaster occurred, residential environment (home residents or facility residents), whether a participant used facilities that were shut down after the disaster, whether a participant was recertified and population density. We defined facilities that were shut down after the disaster as any care service that had users before the disaster and then changed status to having no users after the disaster during the observation period.

### Ethical approval

Ethical approval was granted by the Ethics Committee for Epidemiological Research at Hiroshima University (Ref. no. E-1389).

### Statistical analysis

We showed the baseline characteristics of victims and non-victims. We used a chi-square test for the discrete variables and Wilcoxon’s rank-sum test for ordinal variables and for continuous variables without a normal distribution.

Survival analysis was done using Kaplan-Meier analysis and a log-rank test to estimate the disaster risk. “Month = 0” was July 2018: which was the start of the assessment when the 2018 Japan Floods occurred. The Cox proportional hazards model was used to assess cognitive decline. To examine the effect of the disaster, multivariate analysis was conducted adjusting for age classification, gender, level of dementia scale before the disaster occurred, residential environment (home residents or facility residents), whether a participant used facilities that were shut down after the disaster and population density in addition to the crude model. After we confirmed that the interaction term between whether the users were victims and residential environment (home residents or facility residents) was statistically significant (*p* = 0.017), we stratified them for the analysis. Furthermore, we confirmed that there were no significant interaction terms between whether the users were victims and other covariates, including age classification.

In addition, we performed two sensitivity analyses. The first sensitivity analysis was the same Cox proportional hazards model restricted to only re-certified participants as in Sensitivity analysis 1. Sensitivity analysis 2 was the cox proportional hazards model restricted to only participants who were aged 85 years or older and we stratified the age code more precisely: 85–89, 90–94, and over 95. Cognitive function can rapidly decline in persons over 85 years old [31]. Because the proportion of people aged over 85 was higher in non-victims than victims, we conducted this analysis.

After a disaster occurs, simple estimation is important to approach a high-risk population. Therefore, we conducted a sub-group analysis. The residential environment (home or facility) and the level of DSA before the disaster were used for this grouping. Because a DSA level of 2b or lower allows for independent living with or without care support, the DSA level of 2b was adopted as the cutoff point. The four groups were as follows: (1) home residents who could live independently for the most activities of daily living with or without any care support (level of DSA ≤ IIb), (2) home residents who could not live independently for most activities of daily living without constant care support (level of DSA ≥ IIIa), (3) facility residents who could live independently for most activities of daily living with or without any care support (level of DSA ≤ IIb), and (4) facility residents who could not live independently for most activities of daily living without constant care support (level of DSA ≥ IIIa). We examined the hazard ratio of cognitive decline by the disaster in each group. The reference was non-victims in each group. After the Cox proportional hazard models, we confirmed the proportionality of the hazard assumption.

We performed all statistical analyses using STATA/MP version 16 (StataCorp, 2019).

## Results

The total number of participants was 264,614. Victims accounted for 1.10% of the total participants (*n* = 2,908). We show participant characteristics in Table 1. The proportion of males was around 30% in both groups. The proportion of DSA level before the disaster was lower in victims than non-victims by chi-squared test (*p* < 0.001). Facility residents were fewer in victims than non-victims (*p* < 0.001). Victims more often used facilities that were shut down after the disaster (*p* < 0.001). Victims received more re-certification for care level after the disaster (*p* < 0.001). Population density was lower in victims than non-victims (*p* < 0.001). The prevalence of cognitive decline was 294 (10.11%) in victims and 23,389 (8.94%) in non-victims (*p* < 0.031).

**Table 1:** Demographic characteristics

		Disaster victims		Non-victims of the disaster		*p* value
		*n* = 2908		*n* = 261,671		
Age, no. (%)	Under 65	56	(1.93)	4466	(1.71)	0.019^a^
	65–74	298	(10.25)	25,264	(9.65)	
	75–84	979	(33.67)	82,661	(31.59)	
	over 85	1575	(54.16)	149,280	(57.05)	
	85–89	825	(28.37)	72,873	(27.85)	0.017^a^
	90–94	529	(18.19)	53,322	(20.38)	
	over 95	221	(7.60)	23,085	(8.82)	
Gender, no. (%)	Male	874	(30.06)	75,345	(28.79)	0.135^a^
	Female	2034	(68.94)	186,326	(71.21)	
Level of dementia symptomatology assessment before disaster, no. (%)	Independent	445	(15.30)	37,792	(14.44)	< 0.001^a^
	I	574	(19.74)	47,321	(18.08)	
	II a	381	(13.10)	33,152	(12.67)	
	II b	575	(19.77)	50,756	(19.40)	
	III a	467	(16.06)	47,708	(18.23)	
	III b	203	(6.98)	18,638	(7.12)	
	IV	263	(9.04)	26,304	(10.05)	
Facility residents, no. (%)		655	(22.52)	78,826	(30.12)	< 0.001^a^
Use of facilities that were shut down after the disaster		652	(22.42)	8995	(3.44)	< 0.001^a^
Population density (per 1000/km^2^), median (IQR)^d^		1.45	(0.57–1.86)	1.65	(0.70–1.89)	< 0.001^b^
Care level re-certification, no. (%)		1,136	(39.1)	93,519	(8.92)	< 0.001^a^
Cognitive decline, no. (%)		305	(10.5)	23,349	(24.5)	0.003^a^
1 level down, no. (%)		145	(4.99)	11,626	(4.44)	0.27^a^
2 level down, no. (%)		83	(2.85)	6562	(2.51)	
3 or more level down, no. (%)		77	(2.65)	5161	(1.97)	
Observation period, median (IQR)		6	(6–6)	6	(6–6)	0.27^c^
Incident rate (95% confidential interval)		0.018	(0.016–0.020)	0.016	(0.016–0.016)	

^a^Chi-squared test, ^b^Wilcoxon rank-sum test, ^c^Kruskal-Wallis test

^d^The data on population density in each municipality extracted from the Statistics Bureau, Ministry of Internal Affairs and Communications [24]

**Figure 2:**
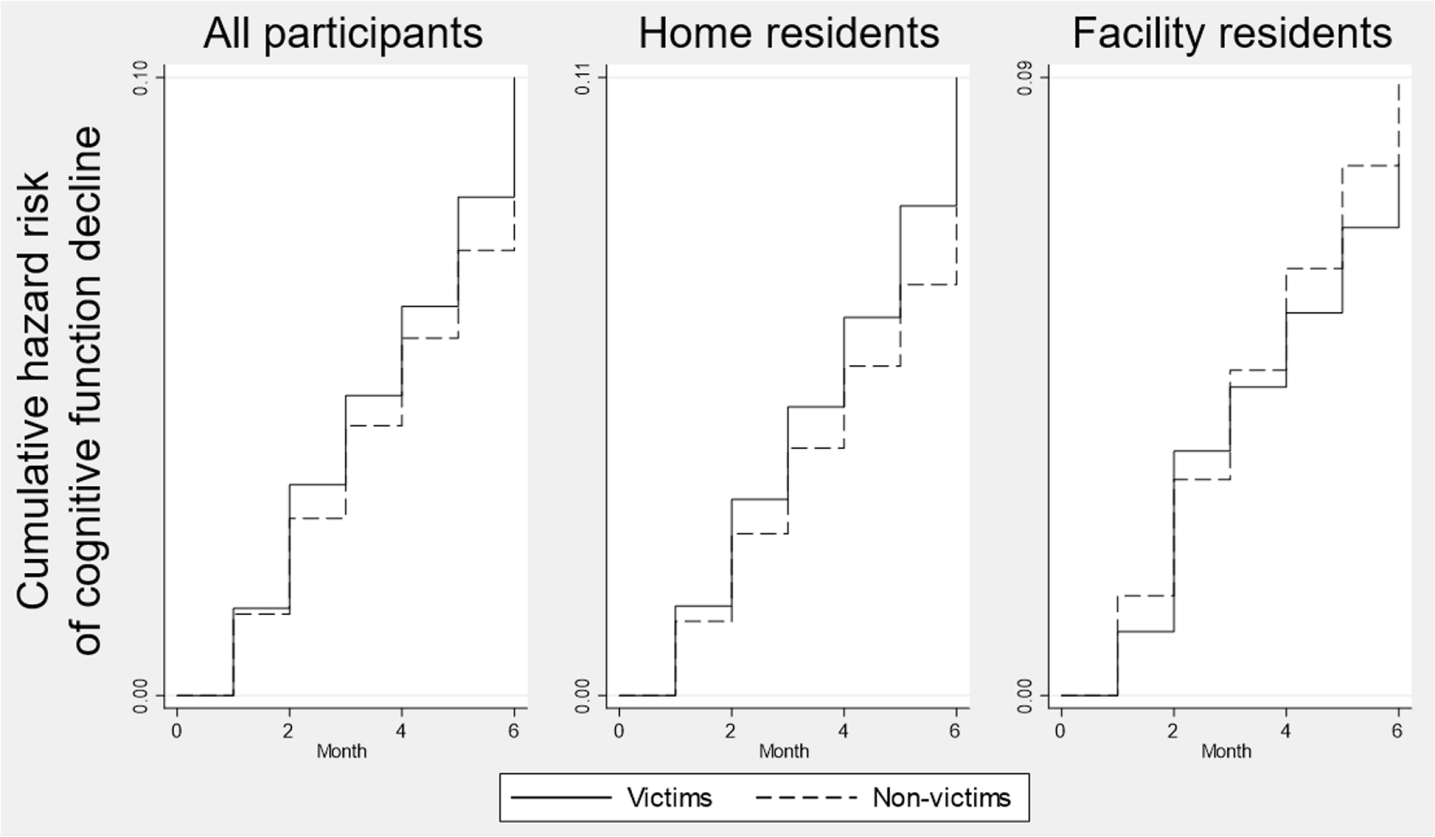
Kaplan-Meier failure curves from time of disaster occurring by home residents and facility residents. Footnote: Home residents were users who did not use facility service until the disaster

Figure 2 shows the Kaplan-Meier survival curves from the time of disaster occurring by home residents and facility residents. The total observation period was up to 6 months. Cognitive function of victims among home residents declined more than non-victims by log-rank test (*p* = 0.002). There is no significant difference between victims and non-victims in facility (*p* = 0.242).

We conducted Cox regression analysis for the impact of the disaster. In the crude model, the hazard ratio of the victims was 1.18 (95% confidential interval (CI): 1.05-1.32). We show the results of multivariate Cox regression analysis in Table 2. Because the *p* value of interaction term between whether a participant was a victim and residential environment was 0.017, we added the model stratified by residential environment. The hazard ratio of the impact of the disaster among the home residents was 1.20 (95% CI: 1.06–1.36). The hazard ratio among the facility residents was 0.89 (95% CI: 0.67–1.17). Sensitivity analysis 1, in which the analysis was restricted to only those who had been recertified, showed similar trends. The hazard ratio of the impact of the disaster among the home residents was 1.13 (95% CI: 1.00–1.28). The hazard ratio among the facility residents was 0.91 (95% CI: 0.69–1.21). The same trend was observed in Sensitivity analysis 2, which focused only on those aged 85 years and older (Table 3). The hazard ratio for the impact of the disaster among the home residents was 1.23 (95% CI: 1.04–1.45). The hazard ratio among the facility residents was 0.91 (95% CI: 0.60–1.20).

**Table 2:** Multivariate Cox regression analysis for the impact of disaster on cognitive decline

		Whole participants						Re-certified participants					
		All		Home residents		Facility residents		All		Home residents		Facility residents	
		H.R.	95% C.I.	H.R.	95% C.I.	H.R.	95% C.I.	H.R.	95% C.I.	H.R.	95% C.I.	H.R.	95% C.I.
Victims (ref = non-victims)		1.12	1.00^a^–1.26	1.20	1.06–1.36	0.89	0.67–1.17	1.08	0.97–1.21	1.13	1.00^b^–1.28	0.91	0.69–1.21
Age (ref = < 64)	65–74	1.65	1.43–1.91	1.69	1.44–1.99	1.36	0.98–1.89	1.63	1.41–1.88	1.68	1.44–1.99	1.22	0.88–1.70
	75–84	2.16	1.88–2.48	2.29	1.96–2.67	1.48	1.08–2.04	2.09	1.82–2.41	2.26	1.93–2.64	1.28	0.93–1.75
	85 >	2.54	2.20–2.91	2.83	2.42–3.30	1.49	1.09–2.05	2.45	2.13–2.81	2.78	2.38–3.25	1.30	0.94–1.78
Female (ref = male)		0.93	0.91–0.96	0.93	0.90–0.97	0.96	0.91–1.02	0.96	0.93–0.99	0.96	0.93–0.99	0.99	0.94–1.05
Level of dementia symptomatology assessment before disaster (ref = II b)	Independent	1.38	1.32–1.44	1.35	1.29–1.42	1.96	1.77–2.17	1.45	1.39–1.51	1.46	1.39–1.53	1.74	1.57–1.92
	I	1.19	1.14–1.23	1.14	1.09–1.19	1.50	1.39–1.62	1.20	1.15–1.25	1.18	1.13–1.24	1.35	1.25–1.46
	II a	1.33	1.28–1.39	1.33	1.26–1.39	1.35	1.26–1.46	1.36	1.31–1.42	1.39	1.32–1.46	1.31	1.21–1.41
	III a	0.63	0.60–0.66	0.74	0.70–0.78	0.55	0.51–0.58	0.65	0.63–0.68	0.73	0.69–0.78	0.58	0.55–0.62
	III b	0.54	0.51–0.58	0.66	0.60–0.73	0.48	0.43–0.52	0.56	0.52–0.60	0.64	0.58–0.70	0.51	0.46–0.55
	IV	0.12	0.10–0.13	0.18	0.16–0.21	0.09	0.08–0.10	0.13	0.11–0.14	0.18	0.15–0.21	0.10	0.09–0.12
Use of facilities that were shut down after the disaster (ref = none)		1.31	1.23–1.40	1.34	1.24–1.44	1.19	1.05–1.35	1.20	1.13–1.28	1.18	1.09–1.26	1.24	1.10–1.41
Population density (per 1000/km^2^)		0.98	0.97–0.98	0.97	0.96–0.98	1.00	0.99–1.01	1.02	1.01–1.03	1.02	1.02–1.03	1.02	1.00–1.03
Facility residents (ref = home residents)		1.45	1.41–1.50					1.59	1.54–1.64				

*H.R*., hazard ratio; *C.I.*, confidence interval

^a^The lower limit of the 95% C.I. is 1.0018 rounded off to the third decimal

^b^The lower limit of the 95% C.I. is 1.0004 rounded off to the third decimal

**Table 3:** Multivariate Cox regression analysis for the impact of disaster on cognitive decline among participants who are aged over 85

		Whole participants					
		All		Home residents		Facility residents	
		H.R.	95% C.I.	H.R.	95% C.I.	H.R.	95% C.I.
Victims (ref = non-victims)		1.13	0.97–1.31	1.23	1.04–1.45	0.85	0.60–1.20
Age (ref = 85–89)	90–94	1.08	1.04–1.12	1.10	1.05–1.15	1.02	0.96–1.09
	over 95	1.18	1.12–1.23	1.27	1.19–1.36	1.04	0.96–1.12
Female (ref = male)		0.95	0.91–0.98	0.94	0.89–0.98	0.99	0.92–1.06
Level of dementia symptomatology assessment before disaster (ref = II b)	Independent	1.65	1.56–1.75	1.62	1.52–1.73	2.21	1.94–2.51
	I	1.27	1.21–1.33	1.24	1.17–1.31	1.49	1.35–1.64
	II a	1.36	1.29–1.42	1.39	1.30–1.48	1.31	1.20–1.43
	III a	0.61	0.58–0.65	0.72	0.67–0.78	0.53	0.49–0.58
	III b	0.52	0.48–0.57	0.62	0.55–0.71	0.47	0.42–0.52
	IV	0.10	0.09–0.12	0.15	0.12–0.19	0.08	0.07–0.10
Use of facilities that were shut down after the disaster (ref = none)		1.28	1.18–1.39	1.33	1.21–1.46	1.11	0.95–1.30
Population density (per 1000/km^2^)		0.98	0.97–0.99	0.98	0.97–0.99	0.99	0.97–1.01
Facility residents (ref = home residents)		1.36	1.31–1.41				

*H.R.*, hazard ratio; *C.I.*, confidence interval

**Figure 3:**
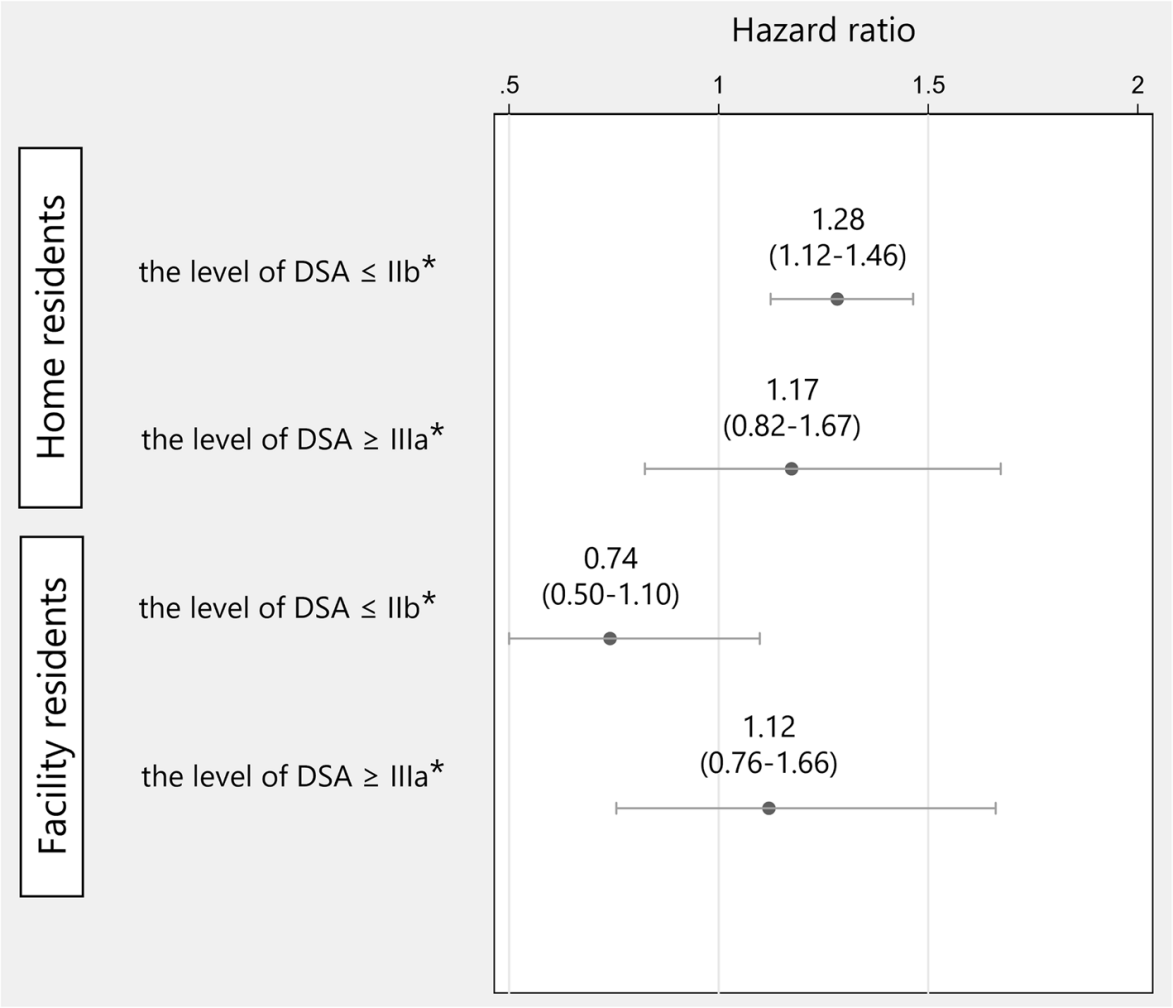
Subgroup analysis by residential environment (home or facility) and the level of dementia symptomatology assessment. *Reference = non-victims. DSA, dementia symptomatology assessment

Figure 3 shows the subgroup analysis results. Persons living at home who could live independently for most activities of daily living with or without any care support (the level of DSA ≤ IIb) were at higher risk of cognitive decline due to the disaster (HR: 1.28, 95% CI: 1.12–1.46). In the other groups, the risk of cognitive decline due to the disaster was not significant.

## Discussion

This study revealed that the 2018 Japan Floods exacerbated a decline in the cognitive function of elderly victims living at home. This is the first study using big data to assess the entire population affected by a natural disaster. Subgroup analysis showed that home residents who could live independently for most activities of daily living with or without any care support (the level of DSA ≤ IIb) were at risk for cognitive decline.

We showed that the 2018 Japan Floods exacerbated the cognitive function of victims among home residents. Participants whose cognitive function was judged to have declined were 10.11% (*n* = 294) among victims and 8.94% (*n* = 23,389) among non-victims. The decline of cognitive function occurred more frequently in victims by chi-squared test (*p* = 0.003). The hazard ratio for home residents was 1.20 (95% CI: 1.06–1.36). The results of Sensitivity analysis 1 and Sensitivity analysis 2 also supported the trend of these results, thus, increasing the robustness. The cognitive decline caused by the disaster was consistent with previous studies on post-disaster cognitive decline (GEJE, Hurricane Katrina and Hurricane Maria) [11–16]. We revealed the same trend in a torrential rain disaster, which has occurred annually in Japan in recent years. In general, it has been reported that mental illness, lack of social contact, and changes in the living environment can exacerbate cognitive function [32]. In natural disasters, mental illness, such as depression and PTSD, and interruption of social activities can also exacerbate cognitive function [12, 33–35].

The participants identified as victims in this study were mainly those whose houses were damaged and/or lost a main breadwinner. The majority evacuated to a shelter, but found it difficult to engage in their usual social activities, or suffered mental stress due to the death of family members. Since we could not distinguish among these actual damages among victims, the results of this study may represent a complex and mixed effect on the cognitive function by the disaster. This study provides a significant novelty in that we could detect almost all victims of a large natural disaster and evaluate the change in their cognitive function. However, the outcome was a relatively short-term result. The long-term effect on cognitive function needs to be evaluated in a future study.

The subgroup analysis showed that home victims who could live independently for most of their activities of daily living with or without any care support (the level of DSA ≤ IIb) were at risk of the cognitive decline due to the disaster. There were two hypotheses for why this may have been the case. First, although some would have had problems with cognitive function before the disaster, they could live independently with or without care support in some way, and thus, the decline in cognitive function had not come to the surface. However, due to the environmental changes and lack of care support caused by the disaster, the problem of cognitive decline surfaced, and their primary care physicians made a determination that their cognitive function had deteriorated. Second, disaster stress impacted cognitive function directly due to environmental changes. It has been reported that stress, such as strong depression, can affect cognitive decline in people with mild cognitive impairment [12]. Decreased physical and social activities have also been reported to contribute to the decline of cognitive function [32]. In whichever case, primary care physicians and caregivers should carefully watch home victims who were relatively independent in terms of cognitive function before the disaster and recognize that the disaster is likely to exacerbate their cognitive function. It is also important to carefully monitor changes in the environment, including family changes and dwellings. In addition, since it is difficult for victims to report a decline of cognitive function on their own, outreach by care professions or local government to check on at-risk persons after the disaster may be helpful in early detection and implementation of countermeasures for cognitive decline [36].

A high age classification was associated with the high prevalence of cognitive decline. In addition, males had a higher risk of cognitive decline than females. It is well known that cognitive function declines with increasing age [37]. The lower risk of cognitive decline from baseline in females may be due to the baseline cognitive function with age. Females were older and more lived in a facility, and their levels of DSA were higher than males. As such, there was a lower prevalence of cognitive decline from before the disaster. The use of facilities that were shut down after the disaster was a risk factor for cognitive decline. In the GEJE, it was reported that the mortality rate of residents who evacuated to different care facilities from their usual care facility was high [38]. One of the reasons was the absence of their usual care. Similarly, the absence of their usual care could have contributed to the exacerbation of cognitive function. In Japanese long-term care settings, information on individualized care is shared in an old-fashioned way and is done orally in meetings and recorded only on each caregiver’s notes. Therefore, if the usual care providers are absent, the information is often completely unknown. The establishment of a system to share such individualized care information is quite important to prepare for in a disaster. It was also found that participants who lived in a municipality with a lower population density had a higher incident ratio of cognitive decline than those in an area with a higher population density. This might represent the lack of long-term care resources and the difficulty in finding alternative LTCI services in rural areas when there were any changes in cognitive function. Furthermore, the reconstruction of roads and utilities was slower in rural areas than in urban areas [39]. In Japan, low population density areas are often rural and have a high aging rate. To decrease disparities in the provision of LTCI services between rural and urban areas, it is necessary to share long-term care resources seamlessly beyond the municipalities.

This was the first study to evaluate for change in cognitive function due to the 2018 Japan Floods using LTCI claim data that covered all LTCI service certified users. Therefore, this study examined the overall effect of this disaster on the cognitive function with LTCI certified users. The database is quite accurate because it is managed by the national government.

This study has several limitations. First, the LTCI database has little information about death and medical attendance, such as diseases, treatment, and admission. Therefore, we could not detect what medical conditions exacerbated the cognitive function of participants. It is also possible that victims were more likely to be assessed for changes in cognitive function by medical providers and caregivers due to a deteriorating health status. The number of recertifications was also higher among victims, but the LTCI database does not include the reasons for why recertification was submitted. Second, some participants were misclassified into non-victims. Welfare recipients and A-bomb survivors could not be classified using the exemption of LTCI service fees. The number of welfare recipients who used LTCI service is 3,953 in the setting area [40]. Similarly, the total number of A-bomb survivors regardless of whether a participant received the certification of LTCI is 17,232 [41]. Since the proportion of victims among all participants was 1.10%, we estimated that the impact of the misclassification is not significant, with about 200 participants misclassified. In addition, there are people who were permitted to delay out-of-pocket payments while using the care services after the disaster, rather than having an exemption. Because these misclassified participants were included as non-victims, we risk underestimating the effect of the disaster.

This study showed that the disaster caused by the torrential rains exacerbated a decline in the cognitive function of residential victims. The risk of cognitive decline was high among residential victims who had previously maintained sufficient cognitive function to live independently with or without some care support. In addition, we found that environmental factors were also related, such as a stoppage in LTCI service and having limited care resources in rural areas. It is necessary to construct a system that detects victims among frail elderly and provide adequate support to maintain their cognitive function.

## Conclusions

This study showed that elderly victims of the 2018 Japan Floods were at risk for cognitive decline. It is necessary to provide support to higher-risk groups after such disasters. Medical providers, care providers, and the local governments should establish a system to check on the cognitive function of elderly victims and provide necessary care support.

## Data Availability

Raw data will not be shared due to restrictions stipulated by the Ministry of Health, Labour and Welfare.

## Abbreviations

LTCI: Long-Term Care Insurance
CI: Confidence Interval
GEJE: Great East Japan Earthquake
ADL: Activity of Daily Living
MHLW: Ministry of Health, Labour and Welfare
DSA: Dementia Symptomatology Assessment
